# Is the number of DNA repair genes associated with evolution rate and size of genomes?

**DOI:** 10.1186/s40246-020-00259-3

**Published:** 2020-03-16

**Authors:** Ion Udroiu

**Affiliations:** Dipartimento di Scienze, Università degli Studi Roma Tre, Viale G. Marconi 446, 00146 Rome, Italy

In a recent article, Voskarides et al. [1] investigated the relationship between DNA repair genes and evolution rates in vertebrates. In the last decade, there was an increase in comparative studies seeking correlation between DNA repair and longevity [2–5]. One of the reasons is also to translate this knowledge to human health and understand phenomena like aging and carcinogenesis.

The authors of the article found that the number of DNA repair genes was linearly related to the genome size and the protein number and that species that evolved through adaptive radiation have more DNA repair genes [1]. In inter-species studies, the use of linear regression and independent *t* test (those used by the authors) poses a serious problem: these tests assume that samples are *independent*, but this is not the case. In fact, the samples have different grades of dependency, which derive from their phylogenetic relationship [6]. This is why all tests should be performed using a phylogenetic correction, like phylogenetic-independent contrast [7]. Concerning the results of the article, the authors found that the number of DNA repair genes was linearly related to the genome size, but they also found that mammals have more DNA repair genes. Since mammals have larger genomes, the linear correlation is simply due to the fact that mammals are on the right of the *x*-axis (more repair genes) and on the top of the *y*-axis (larger genomes). In cases like this, phylogenetically informed analysis could reveal if within the classes and orders the linear correlation actually exists or not.

If these considerations are of general value for inter-species studies, a more specific concern as regards the “number of DNA repair genes” was found in the article. The genome and gene database of NCBI (https://www.ncbi.nlm.nih.gov/) is a very precious resource and, among other things, allows the user to view the list of orthologs of a specific gene. However, if a species does not appear on that list, it does not mean that it does not possess its ortholog. In the Supplementary material of the article [1], *MAD2L2* is noted as absent in *Rousettus aegyptiacus*. Actually, it does not appear on the list of orthologs on NCBI, but it is just because it is registered as *MAD2B* (NCBI gene ID: 107513121), which is a synonym of *MAD2L2*.

Another example is *MPG*, which is listed as absent in *Elephantulus edwardii*. In fact, no gene (either listed with a synonym) can be found on NCBI. However, a BLAST search indicates that gene *LOC102847768* shares 77% identity with *MPG* of *Loxodonta africana*; moreover, it is located between *NPRL3* and *RHBDF1*, exactly as *MPG* in *Loxodonta Africana*, and its product shows the alkyladenine DNA glycosylase domain, exactly like MPG. Therefore, we can say that *LOC102847768* is an ortholog of *MPG*, which therefore should be listed as present in *Elephantulus edwardii*. Besides these examples, it can be noted that many genes listed as absent in the Supplementary material of the article, in reality, are present in the NCBI lists of orthologs, e.g., in *Ornithorhynchus anatinus XRCC1* (NCBI ID: 100086870), *POLA1* (NCBI ID: 103168349), and *BRCA1* (NCBI ID: 103167900). I have not checked all the genes in the Supplementary material, but with ease I found that all the ones listed as absent that I checked were actually present. The only exception is *SLX1B*, which appears to be a human novelty, arising from duplication of *SLX1A*.

Some studies have evidenced the different species-specific efficiency of DNA repair proteins such as 53BP1 [4] and XRCC5 [2] and the expression of DNA repair genes [3], linking them to longevity. It is, on the other hand, very improbable that some vertebrate species do not possess some genes involved in the maintenance of genome integrity, since the basic machinery of DNA repair emerged with eukaryote life and is highly conserved among animals, plants, and fungi [8]. The study on number of genes could be more useful in the field of the response to unrepaired DNA damage (apoptosis, senescence, block), where some hints indicate a greater diversity between taxa [9].

## Data Availability

Not applicable
